# Case Report: A Severe and Multi-Site *Nocardia farcinica* Infection Rapidly and Precisely Identified by Metagenomic Next-Generation Sequencing

**DOI:** 10.3389/fmed.2021.669552

**Published:** 2021-05-24

**Authors:** Mengfan Jiao, Xiang Deng, Hongfu Yang, Junqiang Dong, Jun Lv, Fang Li

**Affiliations:** ^1^Department of Infectious Diseases, The First Affiliated Hospital of Zhengzhou University, Zhengzhou, China; ^2^Precision Medicine Center, Gene Hospital of Henan Province, The First Affiliated Hospital of Zhengzhou University, Zhengzhou, China; ^3^Department of Respiratory and Critical Care Medicine, The First Affiliated Hospital of Zhengzhou University, Zhengzhou, China; ^4^Department of Integrated Intensive Care Unit, The First Affiliated Hospital of Zhengzhou University, Zhengzhou, China; ^5^Department of Imaging and Nuclear Medicine, The First Affiliated Hospital of Zhengzhou University, Zhengzhou, China

**Keywords:** case report, *Nocardia*, metagenomics next-generation sequencing, precise treatment, infectious disease

## Abstract

*Nocardia* genus is an aerobic, gram-positive, and opportunistic pathogen, which mainly affects cell-mediated immunosuppressed patients. Early diagnosis and treatment greatly improve prognosis. However, the limitation of golden standard-bacterial culture exists. Here, we report a 61-year-old male with pneumonia, sepsis and intermuscular abscesses induced by *Nocardia farcinica*. Venous blood culture reported negative results. Former improper diagnosis and treatment did not improve his condition. With the assistant of metagenomic next-generation sequencing, the pathogen was identified as *Nocardia farcinica*. He was then applied with accurate treatment and had a remarkable clinical and radiological improvement.

## Introduction

*Nocardia* genus is an aerobic and gram-positive rod bacterium which belongs to the class: *Actinobacteria*, order: *Actinomycetales*, Family: *Nocardiaceae* and is an opportunistic pathogen (1, 2). *Nocardia* is widely distributed in plants, gardens and soil, and can be divided into more than 80 *Nocardia* species, of which more than 50 species can cause human diseases (3, 4).

Nocardiosis is the infection caused by *Nocardia* species, to which cell-mediated immunosuppressed patients are more susceptible (3, 5). Occasionally, the infection may occur in normal people. Inhalation of a spore or fragment of broken hyphae results in pulmonary nocardiosis, which is the most common primary nocardiosis (6).

Clinical manifestation of nocardiosis is a variable spectrum which makes nocardiosis difficult to diagnose. The traditional diagnosis of Nocardiosis is culture but with some limitations such as limited sensitivity (7). In recent days, a new approach, metagenomic next-generation sequencing (mNGS), is increasingly applied with high sensitivity and specificity and advantages of identification rare and difficult-to-detect pathogens from clinical samples, presenting superiority in infectious diseases (8).

Here we present a case of pneumonia, sepsis, intermuscular abscesses, and pneumonia infected by *Nocardia farcinica* and was identified by mNGS. With the quick and high sensitivity diagnosis method, the patient received prompt treatment and finally recovered.

## Case Description

On October 20, 2020, a 61-years-old male patient from Henan province, China, was admitted to intensive care unit (ICU) of our hospital due to “swelling and pain of skin for 7 days and listless for 1 day.” His blood pressure was 80/55 mgHg with the highest body temperature 38.4°C 1 day before. On examination, his neck, back, and left buttocks were multiple local tissue swelling and significant tenderness, with no skin damage and wound. His families reported no recent injury. He developed edema in his eyelids with a moon face and buffalo back.

Ten days before the admission, the patient had cough and yellow sputum but relieved. The magnetic resonance imaging (MRI) in the local hospital revealed multiple intermuscular abscesses on the neck, back, and left buttocks. The patient received treatment at the local hospital including carbapenem (detailed treatment remained unknown) but with no improvement even severer pain.

The patient had previously been diagnosed with diabetes for 2 months and nephrotic syndrome for 5 months, and had been taken corticosteroid drugs (prednisolone) and tacrolimus for 5 months.

**Figure 1:**
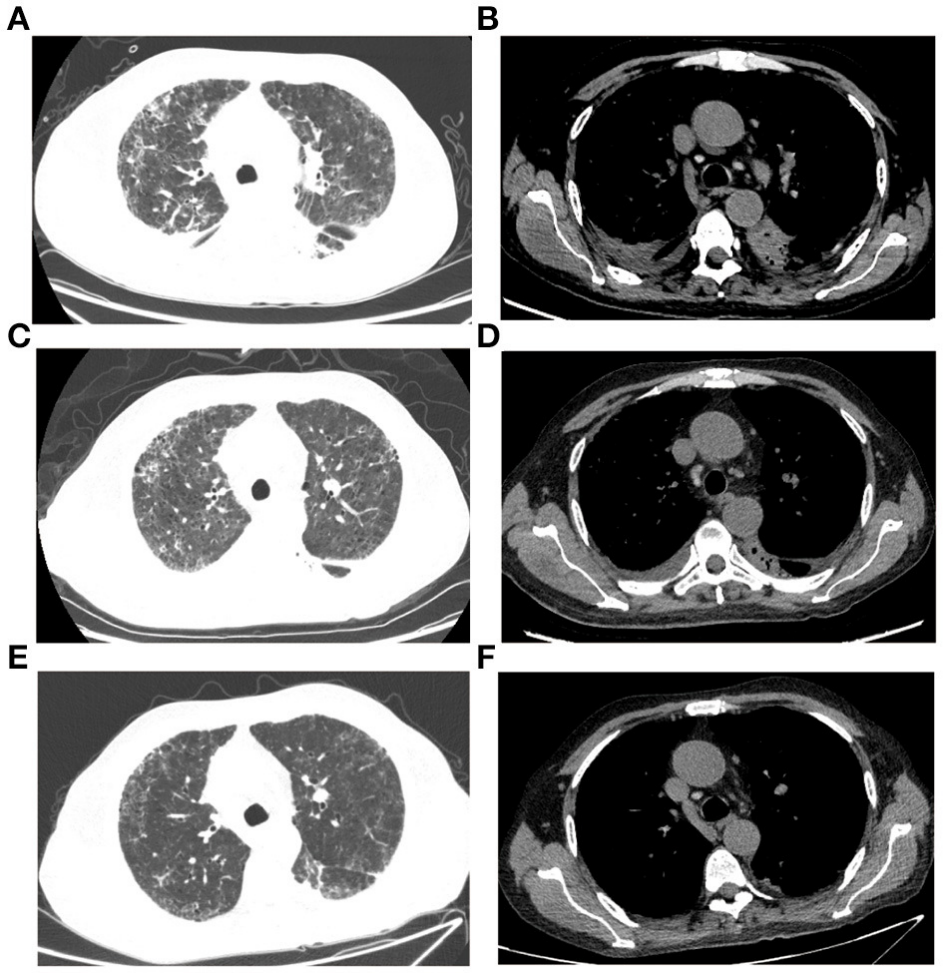
Chest CT imaging of the patient. **(A,B)** CT imaging on October 22, 2020; **(C,D)** CT imaging on November 3, 2020; **(E,F)** CT imaging on December 6, 2020.

The abnormal laboratory test results were as follows: leukocyte count, 14.4 × 10^9^ cells/L; red blood cell count, 3.16 × 10^12^ cells/L; hemoglobin, 95 g/L; neutrophils, 13.42 × 10^9^ cells/L; activated partial thromboplastin time, 19.6 s; fibrinogen, 4.48 g/L; D-Dimer, 3.18 mg/L; fibrin degradation product, 41.32 mg/L; PH, 7.471; total protein, 58 g/L; albumin, 30.5 g/L; Hemoglobin A1C, 9.6%; blood glucose, 13.4 mmol/L; Interleukin 6 8.76 pg/ml. A computed tomography (CT) scan thorax indicated bilateral interstitial pneumonia, bilateral interlobular septal thickening, small nodular shadow and pleural effusion in both lungs (Figure 1). Venous blood cultures for 5 days but reported no bacterial growth. Considering the patient's clinical manifestations, laboratory tests and the history of corticosteroid drugs use, this patient was firstly considered as the infection of opportunistic pathogens. A treatment with linezolid intravenous 0.6 g twice a day and piperacillin/tazobactam intravenous 4.5 g three times a day was initiated to control the infection.

At the same time, the blood sample was collected to perform metagenomic next-generation sequencing (mNGS) on the first day and detected *Nocardia farcinica* on the second day (Table 1). Taking into account the patient's clinical manifestations, history of glucocorticoid use, and the mNGS test results, the patient was diagnosed with *Nocardia* infection. Then anti-infective therapy was changed into compound trimethoprim-sulfamethoxazole (TMP-SMX) tablets 2 pills twice a day, intravenous linezolid, and piperacillin/tazobactam. After consultation in the nephrology department, tacrolimus was discontinued and corticosteroid drugs were gradually discontinued.

**Table 1:** Pathogenic microorganism identified in blood sample.

	**Genus**		**Species**		
**Type**	**Name**	**Sequence number**	**Name**	**Sequence number**	**Relative abundance**
G+	*Nocardia*	188	*Nocardia farcinica*	129	2.667%

Four days after the usage of TMP-SMX, the patient's temperature and leukocyte returned to normal and his symptoms improved significantly.

**Figure 2:**
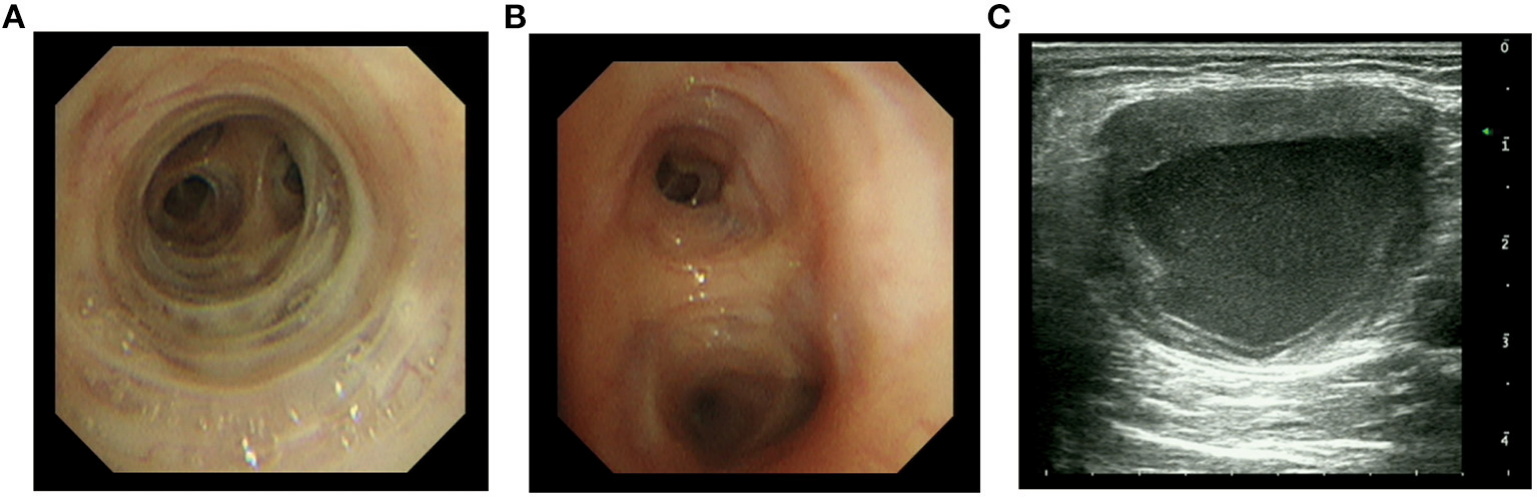
Bronchoscopy and ultrasound imaging of an abscess. **(A,B)** Right middle and left lower bronchoscopy, respectively. **(C)** Ultrasound imaging of an abscess in the left upper arm.

**Figure 3:**
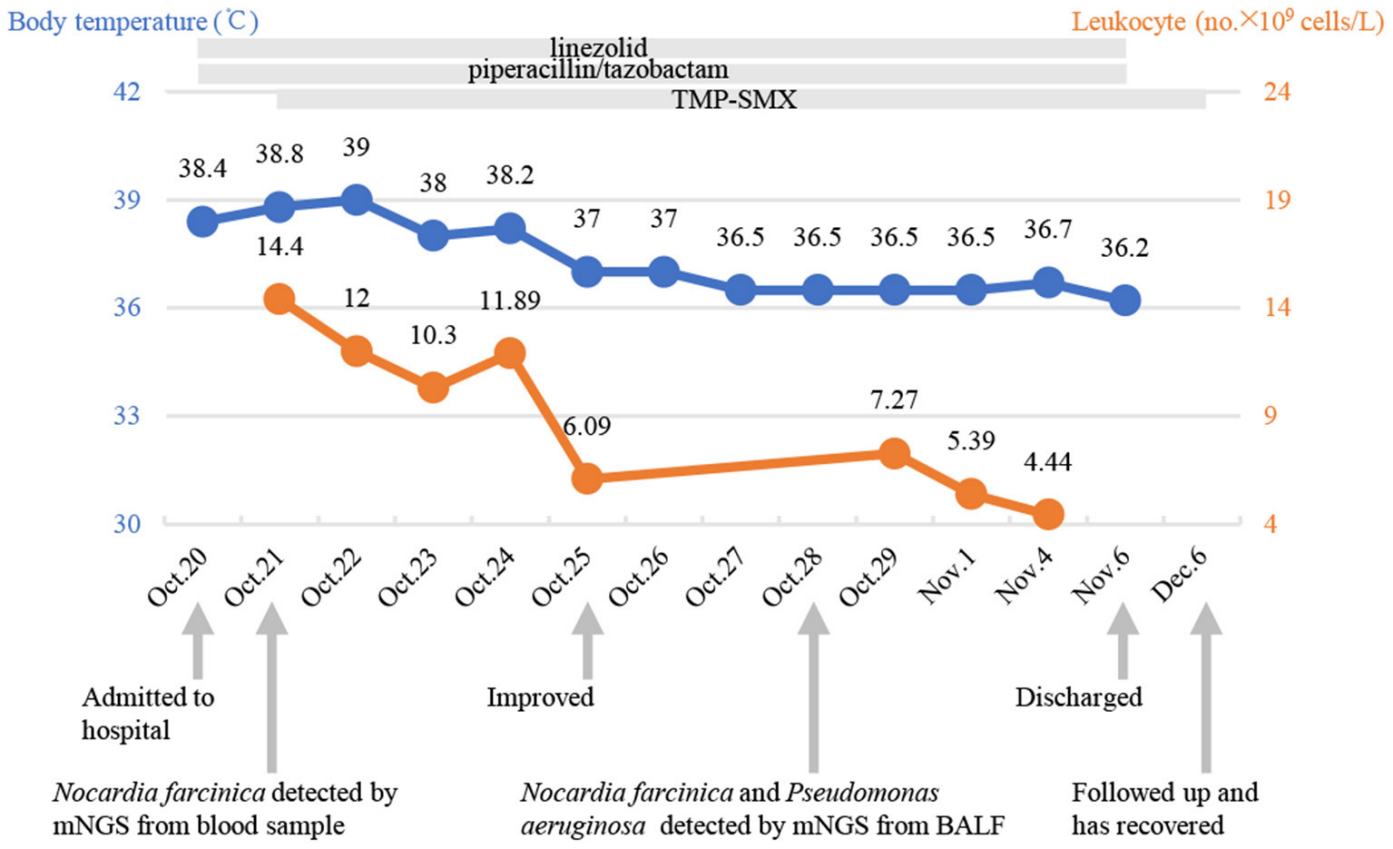
Timeline with relevant data from the case in our hospital; curves of body temperature and leukocyte counts. The arrows below indicate major events. Blue line shows body temperature values. Orange line shows leukocyte counts. TMP-SMX, trimethoprim-sulfamethoxazole; mNGS, metagenomic next-generation sequencing.

On the 7th day, bronchoalveolar lavage fluid (BALF) was collected for mNGS and suggested two bacteria, *Pseudomonas aeruginosa* and *Nocardia farcinica* (Table 2). On the 13th day, bronchoscopy showed that a moderate amount of thin secretion in the bronchus of the lower left lobe (Figure 2). On the 14th day, the puncture was performed under ultrasound guidance (Figure 2). Cyst puncture fluid culture for 2 days reported negative results. The patient's swelling and pain of the skin improved and pulmonary infection and sepsis were gradually under control. His mental state was markedly improved. Also, the laboratory tests and CT scan were in remission (Figure 1). He was discharged after 16 days of treatment. On December 6, 2020, 1 month after the discharge, the patient was admitted again for the review. CT scan showed the disappearance of purulent secretion (Figure 1). He made a great recovery. The patient's timeline was shown (Figure 3).

**Table 2:** Pathogenic microorganism identified in BALF.

	**Genus**		**Species**		
**Type**	**Name**	**Sequence number**	**Name**	**Sequence number**	**Relative abundance**
G–	*Pseudomonas*	621	*Pseudomonas aeruginosa*	503	40.38%
G+	*Nocardia*	17	*Nocardia farcinica*	11	0.745%

## Discussion

The common type species of *Nocardia* include *Nocardia asteroides, Nocardia brasiliensis, Nocardia farcinica, Nocardia otitidiscaviarum*, etc., all of which may cause a variety of diseases in both humans and animals. *Nocardia* is an opportunistic pathogen and mainly affects immunosuppression patients (3, 5), such as patients with acquired immune deficiency syndrome (AIDS) (9), chronic granulomatous disease (10, 11), transplant recipients (12, 13), and with drug-induced systemic immunosuppression (14). It is also found that one-third of nocardiosis patients are immunocompetent (15).

Pulmonary nocardiosis is the most common primary infection, through the inhalation of airborne spores or mycelial fragments from the environment, and is more common in immunosuppression individuals (16). Pulmonary nocardiosis can present as cough, fever, chest pain, and is similar with other pulmonary infection (6). Chest radiographic manifestations are variable, showing as large irregular nodules, nodules, pleural effusions (17), etc., in which pleural effusions account for one-third of all the manifestations (5). Other common *Nocardia* infections have also been reported as central nervous system (CNS) nocardiosis (18) and cutaneous nocardiosis (1, 19) and may be the secondary infection of pulmonary nocardiosis through hematogenous dissemination in immunosuppression patients (20). Primary cutaneous infections tends more common in immunocompetent patient due to the open injury (3). This patient firstly presented respiratory symptoms 10 days prior the admission and then developed skin swelling and pain, when was 7 days before the admission. This patient had taken corticosteroid drugs and tacrolimus for 5 months and he had not suffered open injury. Thus, we considered pulmonary nocardiosis as primary infection for this patient. It would be perfect if the intermuscular abscesses specimen was performed with mNGS. However, the cost of mNGS was a burden to this patient. Moreover, we considered the intermuscular abscesses were the result of primary pulmonary nocardiosis through hematogenous dissemination. Therefore, the necessity of intermuscular abscesses for mNGS could be limited.

The traditional diagnostic method of nocardiosis is culture. A culture of *Nocardia* usually takes 2–7 days, which may delay establishing the diagnosis. Moreover, most patients with nocardiosis may receive antibiotic treatment before samples collection, reducing microbial culture sensitivity (21). In 2005, 16S rRNA–based polymerase chain reaction (PCR) assay was reported to detect *Nocardia* on clinical samples, with high sensitivity and specificity (22), whereas the method requires the pre-considerations of *Nocardia* infection by clinicians. Metagenomic next-generation sequencing (mNGS) is a new approach with characteristics of high sensitivity, rapid detection and less affected by prior antibiotics usage (23, 24). This is an unbiased method and can theoretically detect all kinds of pathogens, which is suitable for difficult and atypical infectious diseases (24). The former study reported mean time to diagnosis was 42 days, which may lead to poor clinical prognosis especially in immunosuppression patients (14). In this case, mNGS only took 2 days to detect pathogens and significantly reduced hospital days. A recent study revealed that 14 samples were identified to be *Nocardia* spp. positive by mNGS, whereas only five of them obtained positive results of culture (25). This patient's negative blood culture may due to the influence of prior antibiotics usage.

mNGS also has its limitation. The expensive cost is a burden to some patients, which was one of the reasons that the intermuscular abscesses specimen was not performed with mNGS in this case. Moreover, the time of sample collection may influence the sensitivity. A former study indicated the false negative of mNGS in *Angiostrongylus cantonensis* detection, which maybe as the result of the improper sampling time during the life cycle of parasites (26). For pulmonary infection, sample types may also influence the sensitivity (27).

Treatment of nocardiosis is individualized (28). Sulfonamides were initially used as nocardiosis treatment (29). The most common treatment for *Nocardia* nowadays is trimethoprim-sulfamethoxazole (TMP-SMX) (5, 29). Linezolid and carbapenem are also used in nocardiosis (7, 30). In this case, previously carbapenem use did not control the infection, whereas treatment of TMP-SMX and linezolid provided symptomatic improvement.

*Pseudomonas aeruginosa* was also detected by mNGS in BALF. Carbapenem was applied at local hospital but without response. It could be deduced that the *Pseudomonas aeruginosa* of the patient responded less sensitive to carbapenem. There was another possibility that *Pseudomonas aeruginosa* was hospital-acquired infections in ICU of our hospital. Before the detection of *Pseudomonas aeruginosa*, the therapy of linezolid, piperacillin/tazobactam and TMP-SMX greatly relieved the patient's symptoms. Actually, linezolid and piperacillin/tazobactam also had clinical efficiency with *Pseudomonas aeruginosa* (31). Therefore, the former treatment was not changed and then patient completely recovered. *Pseudomonas aeruginosa* in this case may be more sensitive to piperacillin/tazobactam, or a combination of these drugs.

## Conclusions

mNGS identified *Nocardia farcinica* of the patient and the appliance of accurate treatment made him complete recovery. mNGS, a new approach with high sensitivity and rapid detection, could assist to identify clinical infection disease pathogens, especially in culture-negative infections. We look forward that mNGS can provide more support for clinical diagnosis and treatment.

## Data Availability Statement

MNGS was performed at Illumina NextSeq 550 platform in Gene Hospital of Henan Province. Metagenomics whole genome shotgun sequences have been uploaded to EMBL. SRA accession: PRJEB43515.

## Ethics Statement

The studies involving human participants were reviewed and approved by the institutional review board of the First Affiliated Hospital of Zhengzhou University. The ethics approval number is 2019-KY-330. The patients/participants provided their written informed consent to participate in this study.

## Author Contributions

MJ, FL, and JL analyzed and interpreted patient data. XD, HY, and JD performed the experiments. MJ and FL analyzed the genomics data. MJ and XD wrote the manuscript. All authors contributed to the article and approved the submitted version.

## Conflict of Interest

The authors declare that the research was conducted in the absence of any commercial or financial relationships that could be construed as a potential conflict of interest.

## References

[1] Mcneil MM Brown JM. The medically important aerobic actinomycetes: epidemiology and microbiology. Clin Microbiol Rev. (1994) 7:357–417. 10.1128/CMR.7.3.357 7923055 PMC358331

[2] Conville PS Witebsky FG. The complexity of nocardia taxonomy: implications for the clinical microbiology laboratory. Clin Microbiol Newslett. (2010) 32:119–25. 10.1016/j.clinmicnews.2010.07.004

[3] Lerner PI. Nocardiosis. Clin Infect Dis. (1996) 22:891–905. 10.1093/clinids/22.6.891 8783685

[4] Rathish BZP. Nocardia. (2020). Available online at: https://www.ncbi.nlm.nih.gov/books/NBK560872/ (accessed September 29, 2020).

[5] Wilson J. Nocardiosis: updates and clinical overview. Mayo Clin Proc. (2012) 87:403–7. 10.1016/j.mayocp.2011.11.016 22469352 PMC3498414

[6] Brown-Elliott B Brown J Conville P Wallace R. Clinical and laboratory features of the *Nocardia* spp. based on current molecular taxonomy. Clin Microbiol Rev. (2006) 19:259–82. 10.1128/CMR.19.2.259-282.2006 16614249 PMC1471991

[7] Welsh O Vera-Cabrera L Salinas-Carmona M. Current treatment for nocardia infections. Exp Opin Pharmacother. (2013) 14:2387–98. 10.1517/14656566.2013.842553 24093436

[8] Han D Li Z Li R Tan P Zhang R Li J. mNGS in clinical microbiology laboratories: on the road to maturity. Crit Rev Microbiol. (2019) 45:668–85. 10.1080/1040841X.2019.1681933 31691607

[9] Long PF. A retrospective study of *Nocardia* infections associated with the acquired immune deficiency syndrome (AIDS). Infection. (1994) 22:362–4. 10.1007/BF01715551 7843819

[10] Dorman SE Guide SV Conville PS Decarlo ES Malech HL Gallin JI. Nocardia infection in chronic granulomatous disease. Clin Infect Dis. (2002) 35:390–4. 10.1086/341416 12145721

[11] Jonsson S Wallace RJ Jr Hull SI Musher DM. Recurrent *Nocardia* pneumonia in an adult with chronic granulomatous disease. Am Rev Respir Dis. (1986) 133:932–4. 3706904

[12] Lebeaux D Morelon E Suarez F Lanternier F Scemla A Frange P. Nocardiosis in transplant recipients. Eur J Clin Microbiol Infect Dis. (2014) 33:689–702. 10.1007/s10096-013-2015-5 24272063

[13] Chouciño C Goodman SA Greer JP Stein RS Wolff SN Dummer JS. Nocardial infections in bone marrow transplant recipients. Clin Infect Dis. (1996) 23:1012–9. 10.1093/clinids/23.5.1012 8922795

[14] Martínez Tomás R Menéndez Villanueva R Reyes Calzada S Santos Durantez M Vallés Tarazona JM Modesto Alapont M. Pulmonary nocardiosis: risk factors and outcomes. Respirology. (2007) 12:394–400. 10.1111/j.1440-1843.2007.01078.x 17539844

[15] Beaman BL Burnside J Edwards B Causey W. Nocardial infections in the United States, 1972-1974. J Infect Dis. (1976) 134:286–9. 10.1093/infdis/134.3.286 789786

[16] Saubolle MA Sussland D. Nocardiosis: review of clinical and laboratory experience. J Clin Microbiol. (2003) 41:4497–501. 10.1128/JCM.41.10.4497-4501.2003 14532173 PMC254378

[17] Feigin DS. Nocardiosis of the lung: chest radiographic findings in 21 cases. Radiology. (1986) 159:9–14. 10.1148/radiology.159.1.3952335 3952335

[18] Anagnostou T Arvanitis M Kourkoumpetis TK Desalermos A Carneiro HA Mylonakis E. Nocardiosis of the central nervous system: experience from a general hospital and review of 84 cases from the literature. Medicine. (2014) 93:19–32. 10.1097/MD.0000000000000012 24378740 PMC4616325

[19] Lopes JO Bassanesi MC Alves SH Salla A Benevenga JP Castro MS. Cutaneous *Nocardia* asteroides infection of nontraumatic origin. Rev Inst Med Trop São Paulo. (1994) 36:403–8. 10.1590/S0036-46651994000500003 7569606

[20] Kontoyiannis DP Ruoff K Hooper DC. Nocardia bacteremia. Report of 4 cases and review of the literature. Medicine. (1998) 77:255–67. 10.1097/00005792-199807000-00004 9715730

[21] Rouzaud C Rodriguez-Nava V Catherinot E Méchaï F Bergeron E Farfour E. Clinical assessment of a *Nocardia* PCR-based assay for diagnosis of nocardiosis. J Clin Microbiol. (2018) 56:e00002–18. 10.1128/JCM.00002-18 29563199 PMC5971555

[22] Couble A Rodríguez-Nava V De Montclos MP Boiron P Laurent F. Direct detection of *Nocardia* spp. in clinical samples by a rapid molecular method. J Clin Microbiol. (2005) 43:1921–4. 10.1128/JCM.43.4.1921-1924.2005 15815019 PMC1081390

[23] Miao Q Ma Y Wang Q Pan J Zhang Y Jin W. Microbiological diagnostic performance of metagenomic next-generation sequencing when applied to clinical practice. Clin Infect Dis. (2018) 67:S231–40. 10.1093/cid/ciy693 30423048

[24] Goldberg B Sichtig H Geyer C Ledeboer N Weinstock GM. Making the leap from research laboratory to clinic: challenges and opportunities for next-generation sequencing in infectious disease diagnostics. mBio. (2015) 6:e01888. 10.1128/mBio.01888-15 26646014 PMC4669390

[25] Weng S-S Zhang H-Y Ai J-W Gao Y Liu Y-Y Xu B. Rapid detection of *Nocardia* by next-generation sequencing. Front Cell Infect Microbiol. (2020) 10:13. 10.3389/fcimb.2020.00013 32133300 PMC7040243

[26] Feng L Zhang A Que J Zhou H Wang H Guan Y. The metagenomic next-generation sequencing in diagnosing central nervous system angiostrongyliasis: a case report. BMC Infect Dis. (2020) 20:691. 10.1186/s12879-020-05410-y 32957922 PMC7507257

[27] Wang Q Wu B Yang D Yang C Jin Z Cao J. Optimal specimen type for accurate diagnosis of infectious peripheral pulmonary lesions by mNGS. BMC Pulm Med. (2020) 20:268. 10.1186/s12890-020-01298-1 33059646 PMC7566056

[28] Muñoz J Mirelis B Aragón LM Gutiérrez N Sánchez F Español M. Clinical and microbiological features of nocardiosis 1997-2003. J Med Microbiol. (2007) 56:545–50. 10.1099/jmm.0.46774-0 17374898

[29] Mcneil MM Brown JM Hutwagner LC Schiff TA. Evaluation of therapy for *Nocardia* asteroides complex infections. Infect Dis Clin Pract. (1995) 4, 287–92. 10.1097/00019048-199507000-00011

[30] Moylett EH Pacheco SE Brown-Elliott BA Perry TR Buescher ES Birmingham MC. Clinical experience with linezolid for the treatment of nocardia infection. Clin Infect Dis. (2003) 36:313–8. 10.1086/345907 12539073

[31] Driscoll JA Brody SL Kollef MH. The epidemiology, pathogenesis and treatment of pseudomonas aeruginosa infections. Drugs. (2007) 67:351–68. 10.2165/00003495-200767030-00003 17335295

